# Large Group Housing Systems in Fattening Bulls—Comparison of Behavior and Performance

**DOI:** 10.3389/fvets.2020.543335

**Published:** 2020-12-09

**Authors:** Laura Schneider, Nina Volkmann, Birgit Spindler, Nicole Kemper

**Affiliations:** Institute for Animal Hygiene, Animal Welfare and Animal Behavior, University of Veterinary Medicine Hannover, Hannover, Germany

**Keywords:** fattening cattle, group size, behavioral synchronization, feeding behavior, lying behavior, housing recommendations

## Abstract

According to international housing recommendations, fattening bulls should not be housed in groups of more than 12–20 animals. However, there are no scientific studies supporting these recommendations as most studies on fattening cattle refer to smaller groups. Therefore, the aim of this study was to analyze and compare behavior and performance of 187 fattening bulls housed in different group sizes of 16, 22, and 33 animals. Behavioral observations were performed during three observation periods at an average age of 8.5, 13, and 17 months. Furthermore, body condition, health status and carcass weights were analyzed. Effects of increasing group size were observed regarding more synchronized lying behavior, longer lying durations and more undisturbed feeding and lying behavior. Interindividual variations in lying and feeding as well as mean and maximum percentages of animals participating simultaneously in interactions did not increase with group size. Health and growth performance were satisfactory in all group sizes. Therefore, the results of this study do not provide scientific evidence for the common argument that increasing group size leads to increased aggression. Furthermore, these findings indicate large group systems to be suitable for the housing of fattening cattle and to contribute to increasing animal welfare. Consequently, current recommendations should be revised.

## Introduction

Fattening cattle in Germany are commonly housed in groups of 6–15 animals (1). There is no legislation concerning group size, but international housing recommendations stipulate that fattening bulls should not be housed in groups >12 (2), 15 (3) or 20 animals (4). However, there are no scientific studies supporting these recommendations. The report by the Scientific Committee on Animal Health and Animal Welfare [(5), confirmed by EFSA (6)] confirms this, claiming there is little information available on maximum group size in cattle, and the optimum group size still needs to be determined.

Under natural conditions, group size is self-regulated as animals join a group or leave it depending on the environmental conditions (7). However, housed farm animals lack the possibility of abandoning their group when conditions are adverse, and may possibly suffer from negative effects provoked by unsuitable group sizes like, e.g., restricted access to or increased competition for resources like food, water, comfortable lying places or reduced freedom of movement (7). Therefore, scientific knowledge on the effects of group size in cattle is needed to evaluate existing recommendations or initiate their revision.

A general view possibly responsible for the recommendations mentioned above is that the ability of animals to recognize all group members decreases with increasing group size (5, 7, 8). Consequently, the establishment and maintenance of a stable hierarchy might be restricted, thus leading to increased aggression. Nonetheless, according to Fraser and Broom (9), cattle are able to recognize up to 50–70 individuals while SCAHAW (5) estimates that in groups of more than 40 cattle, problems will arise in establishing social structures. These figures clearly exceed the recommendations mentioned above and, therefore, cannot be used as scientific evidence. Furthermore, the linear increase in aggression with increments of group size was rejected by several studies on poultry (10–13) and pigs (14–16). In cattle, to the best of our knowledge, there is only one previous study evaluating the effects of group size in a large number of animals (17). However, this study is hardly comparable to fattening cattle as the observed animals were older than 2 years and housed in mixed-sex groups. Further studies refer to smaller groups of at maximum 16 animals and vary regarding their outcome on the behavioral effects of group size (18–22).

A hypothesis challenging the increase in aggression with group size is the social tolerance hypothesis introduced by Estevez et al. (10). According to this hypothesis, for animals in large groups with unlimited access to food and water it is uneconomical to expend energy in defending resources from others, as the number of competitors is high and a depletion of resources by others entails minimal costs. Consequently, costs of defending resources exceed possible benefits (23). A more tolerant social system offers an improved freedom of movement, unrestricted by agonistic encounters, and minimal expenditure of time and energy in agonistic behavior enabling the animals to spend more time in exploiting the available resources (7). Consequently, access to resources in large groups is not impaired by agonistic behavior or resource monopolization by despotic animals as long as food is abundant. As housing systems for fattening cattle generally imply an ad libitum feeding regimen ensuring constant feed availability, large group systems seem to be suitable for housing fattening cattle.

Another benefit of housing systems with large groups derives from the hypothesis of larger pens providing more free space in total, even if the number of animals per square meter is kept at a constant level (22, 24, 25). This can be explained by the possibility given to group-housed animals to share their available free space for movement. This hypothesis was initially introduced in pigs, but a study by Telezhenko et al. (22) supported its relevance in cattle. The increasing amount of free space may facilitate the possibility of spatial separation of functional areas in large groups contributing to improved animal welfare. In typical pens for cattle, active animals tend to be in the front part of the pen containing food and water, while lying animals are more likely to retreat to the back part of the pen. With increasing group and pen size, the distance between the front and back part of the pen increases, too, possibly leading to fewer disturbances between animals displaying different activities.

The aim of this study was to analyze and compare behavior and performance of fattening bulls housed in different group sizes including large groups of 22 and 33 animals for the first time. We hypothesized that bulls in large groups have a more tolerant social system, resulting in no individuals suffering from restricted access to resources. Consequently, with increments of group size, no increased level of aggression has to be expected and the lying and feeding behavior of the animals should not be impaired or characterized by increasing interindividual variation within groups. Furthermore, we expected a decreasing number of feeding and lying bouts as well as increasing bout lengths with increments of group size, as the occurrence of disturbances is reduced due to the spatial separation of functional areas. By including two different large group sizes with 22 and 33 animals, we wanted to investigate whether behavior and performance of bulls varied between different group sizes exceeding the recommended values. Confirmation of the hypotheses mentioned above may lead to modifications in the housing recommendations for fattening cattle. Additionally, in the long run, it may contribute to creating behaviorally more appropriate housing systems and, therefore, improvements in animal welfare.

## Materials and Methods

For this study, no ethical approval had to be obtained because it neither involved a prospective evaluation nor laboratory animals and only non-invasive procedures were applied. The study was carried out in accordance with German legislations, the German Animal Welfare Act [German designation: TierSchG; (26)], National Requirements for Animal Husbandry [German designation: TierSchNutztV; (27)], the Animal Protection Guideline for Fattening Cattle of Lower Saxony, Germany (1) as well as the Council of Europe Convention on the Protection of Animals kept for Farming Purposes and its recommendations concerning cattle (4). The study was reviewed and it received approval from the Animal Welfare Officer of the University of Veterinary Medicine Hannover, Foundation (TVO-2017-B5).

### Animals, Housing, and Management

The study was conducted on 187 Simmental bulls housed on two commercial fattening farms in Lower Saxony (farm 1; 308 fattening bulls in total) and North Rhine-Westphalia (farm 2; 700 fattening bulls in total), Germany. The bulls were housed in straw-bedded pens in groups of 22 animals (G22) on farm 1 and 16 (G16) or 33 animals (G33) on farm 2 (Table 1). The space allowance per bull varied on farm 1 from 3.5 m^2^ at the beginning to 4.4 m^2^ at the end of the fattening period. On farm 2, the space allowance was 4.5 m^2^ during the entire fattening period (Table 1). The bulls were fed a total mixed ration (TMR) twice a day. In addition, feed was manually pushed up toward the feed barrier several times a day and residues were removed manually once per day. The feeding area was not provided with feeding gates or head barriers, but with horizontal metal tubes over the bulls' heads (Supplementary Figure 1). With 75 cm manger space per bull, complying with recommendations for fattening bulls weighing more than 650 kg (1), the animal/feeding-place ratio was ~2:1 in all groups (7.7 feeding places in G16, 12.8 feeding places at the beginning and 15.6 feeding places at the end of fattening in G22 and 15.2 feeding places in G33). The feed composition and particle size distribution of the diet are indicated in Table 1. Water was available ad libitum via two drinking troughs per pen located on both sides in the middle of the pen. In G33, two additional drinking troughs were located in the center of the pen. Fresh straw was distributed daily with a straw blower.

**Table 1:** Housing information and ingredients, chemical composition [related to dry matter (DM)] and particle size distribution [determined with a forage particle separator (28)] of the total mixed ration (TMR) for bulls.

**Farm information**	**Farm 1**	**Farm 2**
Number of bulls per group	22^1^	16, 33
Space allowance per bull (m^2^)	3.5/4.4^2^	4.5
Number of feeding places	12.8/15.6	7.7, 15.2
Number of groups observed in this study	4	2, 2
**Ingredients of the TMR (%)**	**Farm 1**	**Farm 2**
Maize silage	85.5	84.1
Concentrated feed/grain	8.4	7.7
Potatoes	4.5	–
Grass silage	0.9	7.7
Mineral and vitamin mix	0.7	0.6
**Chemical composition of the TMR**	**Farm 1**	**Farm 2**
Dry matter (%)	35.9	44.5
Crude protein (% DM)	10.5	11.6
Crude ash (% DM)	3.8	6.0
Crude fat (% DM)	3.2	2.1
Crude fiber (% DM)	16.3	14.2
Nitrogen-free extractives (% DM)	66.2	66.1
**TMR Particle size distribution (%)**	**Farm 1**	**Farm 2**
Particles retained by the 19 mm sieve (long)	3.8	7.9
Particles retained by the 8 mm sieve (medium)	57.6	58.4
Particles on the bottom pen (short)	38.6	33.7

1 *One of the G22-groups consisted of 23 animals in observation period (OP) 1. After OP1, one animal was removed from that group*.

2 *The space allowance on farm 1 increased during fattening as the bulls were transferred to larger pens between OP2 and OP3*.

The animals arrived at the farms at about 6 months of age, originating from different farms, and were assigned to groups remaining constant until the end of fattening. The assignment to the groups was performed considering the animals' body weight with the aim of obtaining homogeneous groups of similar average initial weight. For the study, four groups of 22 animals (G22) and two groups of 16 and 33 animals each (G16 and G33) were selected. One of the G22-groups consisted of 23 animals in observation periods (OP) 1. After OP1, one animal was removed from that group. The mean age of the bulls at slaughtering was 517.4 ± 10.6 days [mean ± standard deviation (SD)] in G16, 562.3 ± 29.2 days in G22 and 532.5 ± 21.7 days in G33. Carcass weights and pathological findings from carcass evaluations (e.g., lesions, abscesses) were provided by the slaughterhouse.

### Body Condition and Health Scoring

Data acquisition began when the animals' arrival at the farms and their assignment to the groups dated back at least 4 weeks. It was performed during three OP at an average age of 8–9 months (OP1), 13 months (OP2) and 17 months (OP3). One of the G33 was only observed in OP2 and OP3. At the beginning of each OP, all animals were scored individually for body condition and health status by one trained observer. The bulls' body condition was assessed following the body condition score (BCS) system described by Edmonson et al. (29), with scores ranging from 1 (emaciated condition) to 5 (obese condition) in steps of 0.5. Health status was assessed considering the welfare criteria for health assessment in accordance with the Welfare Quality® assessment protocol for cattle [Table 2; (30)]. The analyzed welfare criteria were absence of injuries including lameness and integument alterations, and absence of diseases including coughing, nasal or ocular discharge, hampered respiration, bloated rumen and diarrhea. In addition to observations during scoring, bulls' health was monitored by the farmers. In consultation with their farm veterinarians, they documented any pathological event occurring during the fattening period as well as drug use.

**Table 2:** Welfare criteria concerning health following the Welfare Quality® assessment protocol for cattle (30).

**Welfare criteria**		**Description**
Absence of injuries	Lameness	Abnormality of movement
	Integument alterations	Hairless patches and lesions/swellings
Absence of disease	Coughing	Sudden and noisy expulsion of air from the lungs
	Nasal/ocular discharge	Clearly visible flow/discharge from nostrils/eye
	Hampered respiration	Deep and overtly difficult or labored breathing
	Bloated rumen	“Bulge” between hip bone and ribs on the left side
	Diarrhea	Loose watery manure below tail head on both sides of the tail

### Behavioral Observations

Behavioral observations were performed by analyzing video recordings. The animals were videotaped with one video camera per pen (EQ900F, EverFocus Electronics Corporation, Taipei, Taiwan) and an eight-channel hybrid recorder (AXR-108, Monacor International GmbH & Co. KG, Bremen, Germany). The cameras were located above each pen, capturing the entire pen. Individual animals were identified and listed according to the color and patterns of their fur. The video analyses were performed by one observer using the program Interact (Version 17.0.1.2, Mangold International GmbH, Arnstorf, Germany) for observational research. For the group-based evaluation, the activity of the animals was observed for 48 h per OP using a scan sampling technique (31). In intervals of 2 min from 05:30 to 21:00 h and 10 min during the night (21:00–05:30 h), the number of animals feeding, lying and performing interactions was recorded. At an individual level, the behavior of all animals was scanned at intervals of 10 min from 05:30 to 21:00 h on three consecutive days per OP, using a combination of scan sampling and focal animal sampling (31). The 10-min interval was chosen in accordance with Mitlohner et al. (32) and Endres et al. (33). The recorded and analyzed behavioral patterns were feeding and lying. Lying included bulls that were observed in sternal as well as in total lateral recumbency from the end of the lying-down movement until the end of the standing-up movement. An animal was considered to be feeding when its head was completely behind the feed rail and above the feed while ingesting the feed. Interactions were only analyzed at herd level, and there was no differentiation between different types of interactions. Observed interactions were head-to-head fights and play fights, threads, all kinds of play behavior, displacements, mounting, mounting intention and allogrooming, including sniffing and licking at other animals.

### Behavioral Analysis

In accordance with Endres et al. (33), each behavior was assumed to persist for the entire sample interval. Therefore, the duration of each performed behavioral pattern was calculated by multiplying the number of the correspondent sample intervals by two or ten. For the behavioral observations at herd level, the percentage of animals performing each behavior was averaged for each interval for all days. Furthermore, mean and maximum percentages of animals participating simultaneously in interactions were calculated per group and OP. To assess behavioral synchronization, the percentage of time a certain number or percentage of animals spent feeding or lying was averaged for all groups and days. At an individual level, mean and SD of the time spent feeding and lying per 15.5 h-period were calculated for each animal. Furthermore, mean and SD of the number and duration of lying and feeding bouts per 15.5 h-period were determined.

### Statistical Analysis

Statistical analyses were performed using SAS 9.4 (SAS Institute Inc., Cary, NC, USA, 1999). First, a descriptive analysis was performed to show frequency distributions and averages. Subsequently, the dependent variables of time spent lying and feeding, as well as mean bout length and mean number of lying and feeding bouts per 15.5 h-period were analyzed and tested for normal distribution using histograms and a Shapiro-Wilk test to determine a suitable statistical model for the evaluation. The procedure was repeated with carcass weights and with the dependent variables mean and maximum number of animals performing interactions, as well as the percentages of observation time a certain number or percentage of animals spent feeding or lying. To examine differences between group sizes and observation periods, analyses of variance were performed using generalized linear mixed models (GLIMMIX procedure). Group and number of observed groups were considered main effects, were analyzed for all interactions and were nested in the farm, while observation period was included as random effect. Multiple pairwise comparisons were performed using Bonferroni tests. Correlations between carcass weight and BCS and the dependent variables were tested using Spearman's coefficient of correlation.

## Results

### Health, Body Condition, and Growth Performance

The following health problems were observed during scoring: hairless patches on the neck, forehead and carpal joints in 17 bulls (seven animals from G16, five animals from G22 and G33 each), coughing (six animals from G22) and ocular discharge (one animal from G16 and G33 each). No severe health problems were observed and no specific medical treatments were required throughout the fattening period. Carcass evaluation at the slaughterhouse resulted in one finding (abscess at haunch). The observed BCS values ranged from 2.0 to 4.0. The mean BCS values increased from OP1 to OP3 in all three group sizes (Table 3). Mean carcass weights of the bulls ranged from 391.74 ± 21.07 kg in G16 to 410.62 ± 24.4 kg in G33, these increasing with increments of group size (Table 3). They differed between G16 and G22 (*p* = 0.0329) as well as between G16 and G33 (*p* = 0.0115; Table 4).

**Table 3:** Body condition score (BCS) at the different observation periods (OP) and carcass weight in fattening bulls.

	**Group size**	***n***	**Mean ± SD**	**Minimum**	**Maximum**
BCS OP1	16	32	2.8 ± 0.4	2.0	3.0
	22	89^1^	2.8 ± 0.4	2.0	3.0
	33	29^2^^,^^3^	2.9 ± 0.3	2.0	3.0
BCS OP2	16	32	3.0 ± 0.3	2.5	4.0
	22	88	3.0 ± 0.2	2.0	3.0
	33	63^3^	3.0 ± 0.2	2.0	3.5
BCS OP3	16	32	3.0 ± 0.4	2.5	4.0
	22	88	3.0 ± 0.2	2.5	3.0
	33	66	3.0 ± 0.2	2.5	4.0
Carcass weight (kg)	16	32	391.7 ± 21.1	347.0	434.5
	22	87^4^	407.1 ± 44.9	272.2	583.0
	33	65^4^	410.6 ± 24.4	353.5	464.5
Age at slaughtering	16	32	517.4 ± 10.6	494	540
	22	87^4^	562.3 ±.29.2	481	673
	33	65^4^	532.5 ± 21.7	382	557

*BCS scoring system described by Edmonson et al. (29); SD, standard deviation*.

1 *One of the groups of 22 animals initially consisted of 23 bulls. After the end of OP1, one animal was removed from that group*.

2 *One of the groups of 33 animals was only observed in OP2 and OP3*.

3 *In the groups of 33 animals, body condition could not be rated for all animals in OP1 and OP2*.

4 *Slaughter data from one animal is missing*.

**Table 4:** *p*- and *t*-value statistic of Bonferroni adjustment for multiple comparisons.

**Variable**	**Compared groups**	**DF**	***t***	***p***
Weight	G16 vs. G22	176	−2.2	0.0329
	G22 vs. G33	176	−0.7	0.5151
	G16 vs. G33	176	−2.6	0.0115
Percentage of observation time without animals feeding	G16 vs. G22	38	1.5	0.1525
	G22 vs. G33	38	1.7	0.0925
	G16 vs. G33	38	2.7	0.0099
Percentage of observation time with 61–81% bulls lying simultaneously	G16 vs. G22	38	−0.3	0.7432
	G22 vs. G33	38	−2.9	0.0064
	G16 vs. G33	38	−2.8	0.0077
Percentage of observation time with 81–100% bulls lying simultaneously	G16 vs. G22	38	0.1	0.9369
	G22 vs. G33	38	−1.4	0.1583
	G16 vs. G33	38	−1.2	0.2360
Mean percentage of bulls participating in interactions	G16 vs. G22	38	0.2	0.8275
	G22 vs. G33	38	4.7	<0.0001
	G16 vs. G33	38	4.3	0.0001
Maximum percentage of bulls participating in interactions	G16 vs. G22	38	2.0	0.0547
	G22 vs. G33	38	2.9	0.0067
	G16 vs. G33	38	4.2	0.0002
Lying duration (min)	G16 vs. G22	1,566	−9.0	<0.0001
	G22 vs. G33	1,566	−2.1	0.0338
	G16 vs. G33	1,566	−9.9	<0.0001
Number of lying bouts	G16 vs. G22	1,566	3.7	0.0003
	G22 vs. G33	1,566	−4.1	<0.0001
	G16 vs. G33	1,566	0.2	0.8790
Lying bout duration (min)	G16 vs. G22	1,566	−7.7	<0.0001
	G22 vs. G33	1,566	3.3	0.0009
	G16 vs. G33	1,566	−4.4	<0.0001
Feeding duration (min)	G16 vs. G22	1,566	8.1	<0.0001
	G22 vs. G33	1,566	−10.6	<0.0001
	G16 vs. G33	1,566	−0.9	0.3916
Number feeding bouts	G16 vs. G22	1,566	6.3	<0.0001
	G22 vs. G33	1,566	−3.0	0.0028
	G16 vs. G33	1,566	3.4	0.0007
Feeding bout duration (min)	G16 vs. G22	1,566	2.3	0.0243
	G22 vs. G33	1,566	−11.2	<0.0001
	G16 vs. G33	1,566	−6.7	<0.0001

*G16, groups of 16 animals; G22, groups of 22 animals; G33, groups of 33 animals; min, minutes; DF, degrees of freedom*.

### Behavioral Observations at Herd Level

**Figure 1:**
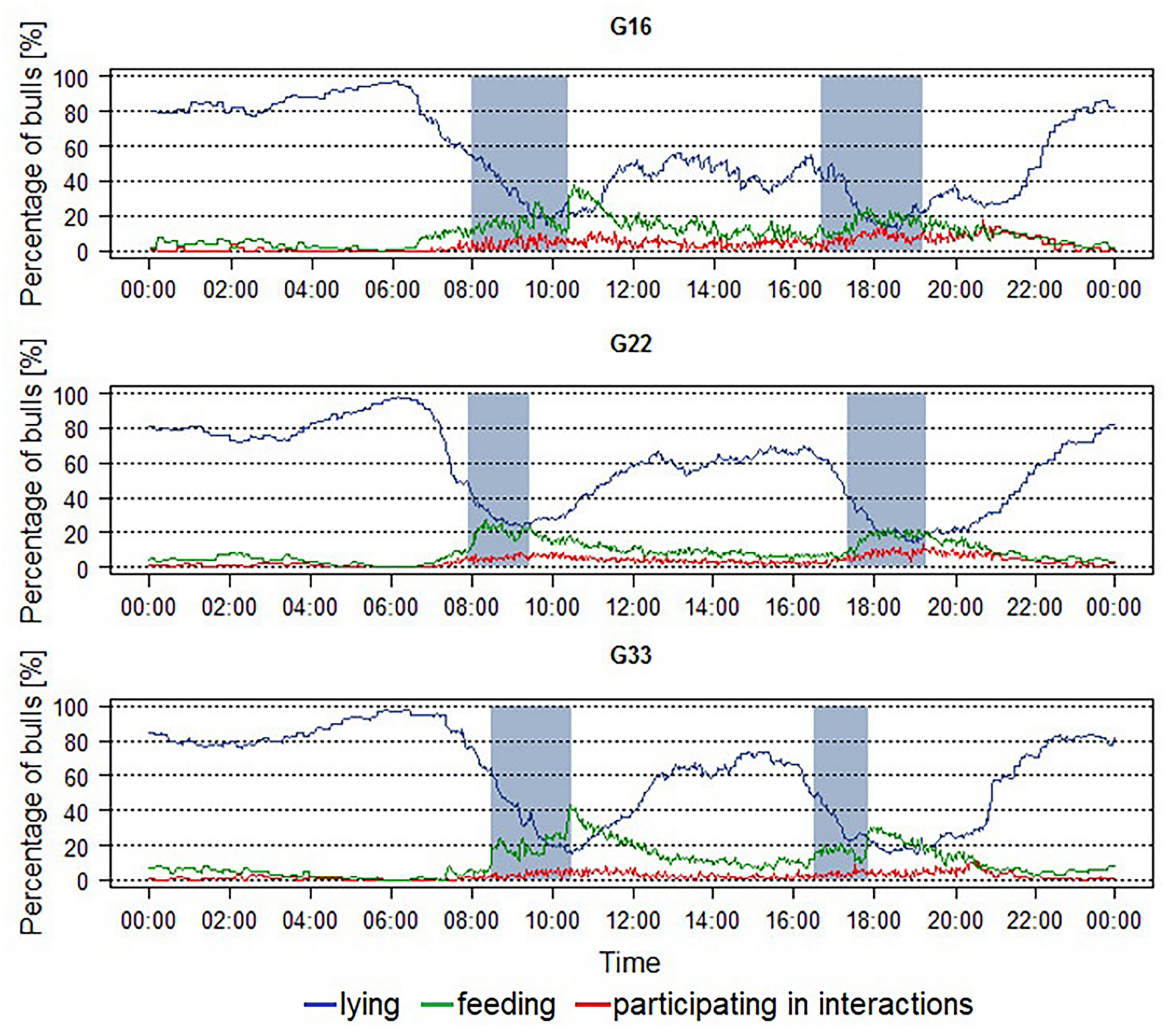
Averaged percentage of fattening bulls feeding, lying and participating in interactions per pen over a 24 h-period in groups of 16 (G16), 22 (G22), and 33 (G33) animals. Blue line = percentage of animals lying, green line = percentage of animals feeding, red line = percentage of animals participating in interactions, blue areas = periods of feed delivery. Data were averaged for each interval for three observation periods of 2 days each and two groups of 16 and 33 animals each as well as four groups of 22 animals.

The averaged percentages of animals feeding, lying, and participating in interactions during the course of the day in the different group sizes are shown in Figure 1. The main lying period with averaged percentages of more than 80% and nearly up to 100% of the animals lying was observed during the night and early morning hours with maximum values occurring around 06:00 h in all groups. Two further lying periods with lower maximum percentages were visible around 13:00 and 16:00 h in all group sizes as well. Minimum percentages of around 20% of animals lying were observed in all group sizes during periods of feed delivery, together with maximum percentages of animals feeding. The feeding activity was widely spread out from morning to evening with averaged percentages of animals feeding rarely exceeding 20%. However, before and shortly after the periods of feed delivery, clear peaks in feeding activity were visible in all group sizes with maximum values at morning feeding exceeding those in the evening. The percentages of bulls participating in interactions also peaked during and after feed delivery in all group sizes. During the night, the percentages approached zero percent.

**Figure 2:**
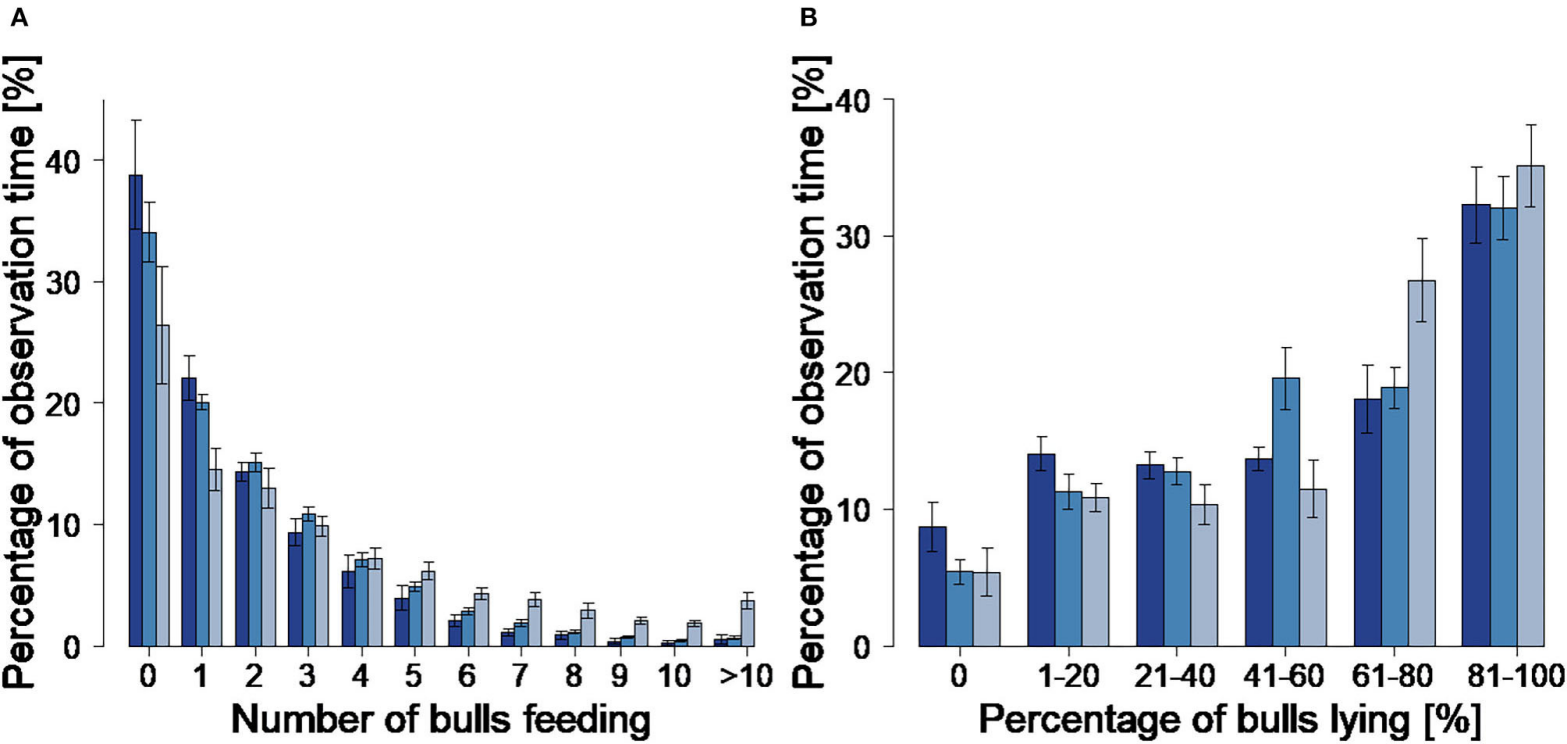
Number of simultaneous feeding animals **(A)** and percentage of simultaneous lying animals **(B)** with corresponding percentage of total observation time. Dark blue = groups of 16 bulls (G16), blue = groups of 22 bulls (G22), light blue = groups of 33 bulls (G33). Bars indicate the mean values, error bars indicate the standard deviations. Data were averaged for three observation periods of 2 days each and two groups of 16 and 33 animals each as well as four groups of 22 animals.

The percentage of observation time without any animals feeding decreased with group size from 38.8 ± 3.6% in G16 to 34.1 ± 0.9% in G22 and 28.0 ± 7.9% in G33 (Figure 2A). This difference was significant between G33 and G16 (*p* = 0.0099; Table 4). The most frequent feeding condition showed one bull feeding alone in all group sizes, followed by two to three bulls feeding at the same time. The higher the number of simultaneous feeding bulls, the lower the averaged percentage of observation time during which the feeding situation was observed. The most frequently observed lying condition was that of 81–100% of the animals lying in all group sizes (Figure 2B). For 61–80% of the bulls lying, the percentage of time it occurred increased with group size, this being higher in G33 than in G 16 (*p* = 0.0077) and G22 (*p* = 0.0064; Table 4). The mean percentage of time that 81–100% of the bulls spent lying simultaneously was highest in G33 with 35.1 ± 7.8%. In the other groups, 81–100% of the animals per pen lying simultaneously occurred during smaller percentages of observation time (32.3 ± 7.4% in G16, 32.1 ± 9.3% in G22). There were no significant differences between the analyzed variables (Table 4).

**Figure 3:**
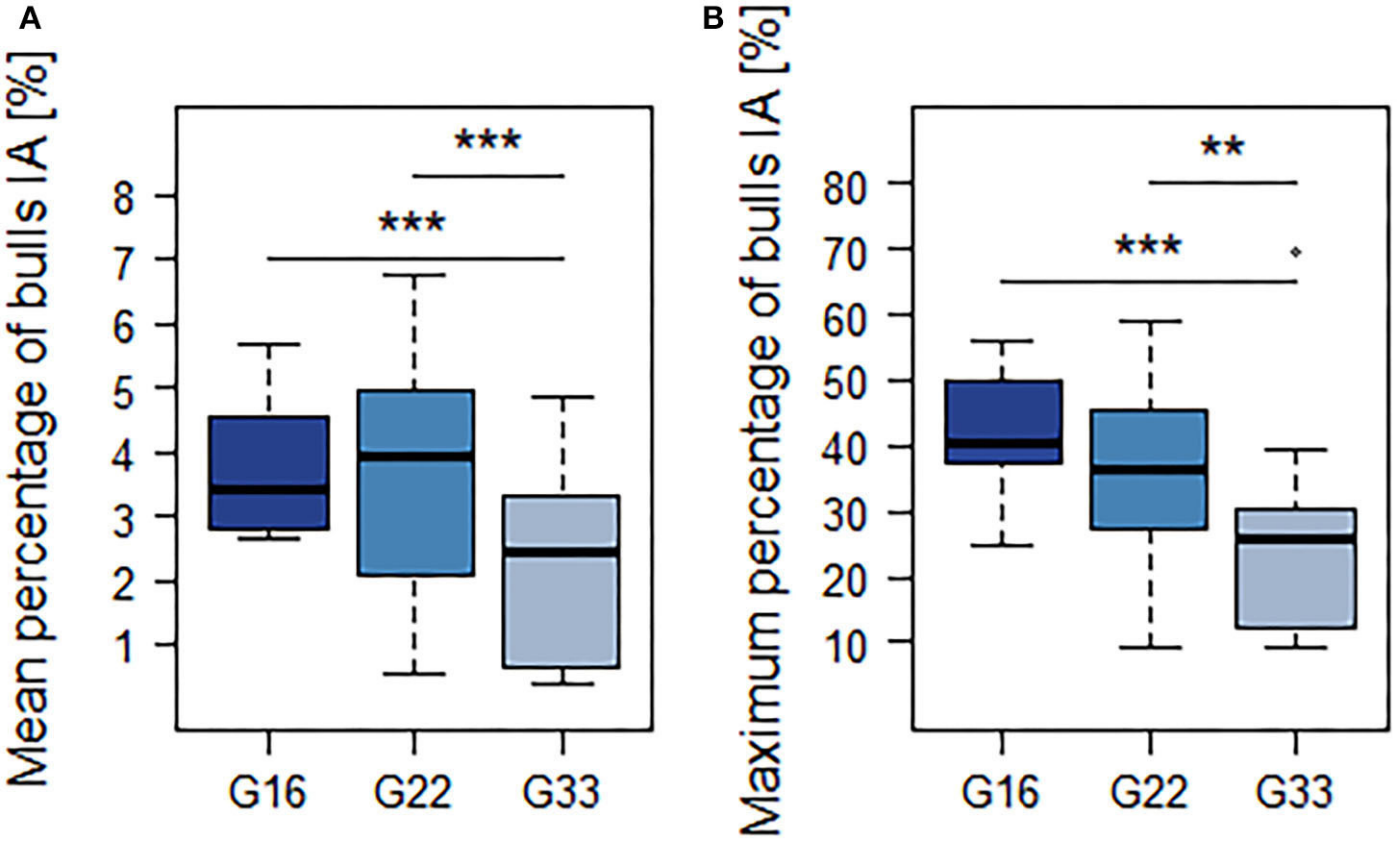
Mean **(A)** and maximum **(B)** percentage of animals participating in interactions. Dark blue = groups of 16 bulls (G16), blue = groups of 22 bulls (G22), light blue = groups of 33 bulls (G33). Data were averaged for three observation periods of 2 days each and two groups of 16 and 33 animals each as well as four groups of 22 animals. **p < 0.01; ***p < 0.001.

Simultaneous lying of all animals per pen (100%) was observed in all group sizes with percentages decreasing with group size (G16: 9.4 ± 5.0%, G22: 7.6 ± 1.3%; G33: 4.7 ± 2.7%). The mean and maximum percentages of animals participating in interactions decreased with group size, but statistically significant differences only occurred between G33 and the smaller group sizes (Figures 3A,B; Table 4).

### Behavioral Observations at Individual Level

**Figure 4:**
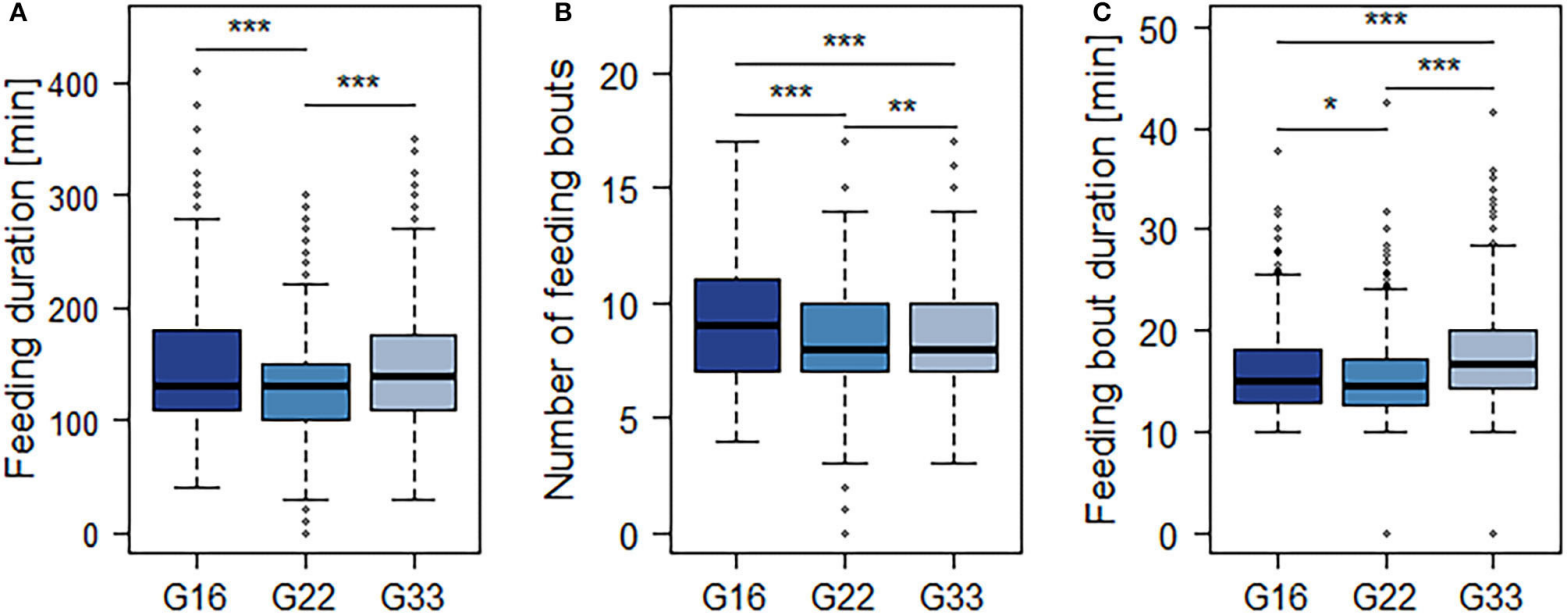
Mean duration of time spent feeding **(A)**, number **(B)** and mean duration **(C)** of feeding bouts per 15.5 h-period. Dark blue = groups of 16 bulls (G16), blue = groups of 22 bulls (G22), light blue = groups of 33 bulls (G33). Data were averaged for three observation periods with three 15.5 h-periods of observation each and two groups of 16 and 33 animals each as well as four groups of 22 animals. *p < 0.05; **p < 0.01; ***p < 0.001.

**Figure 5:**
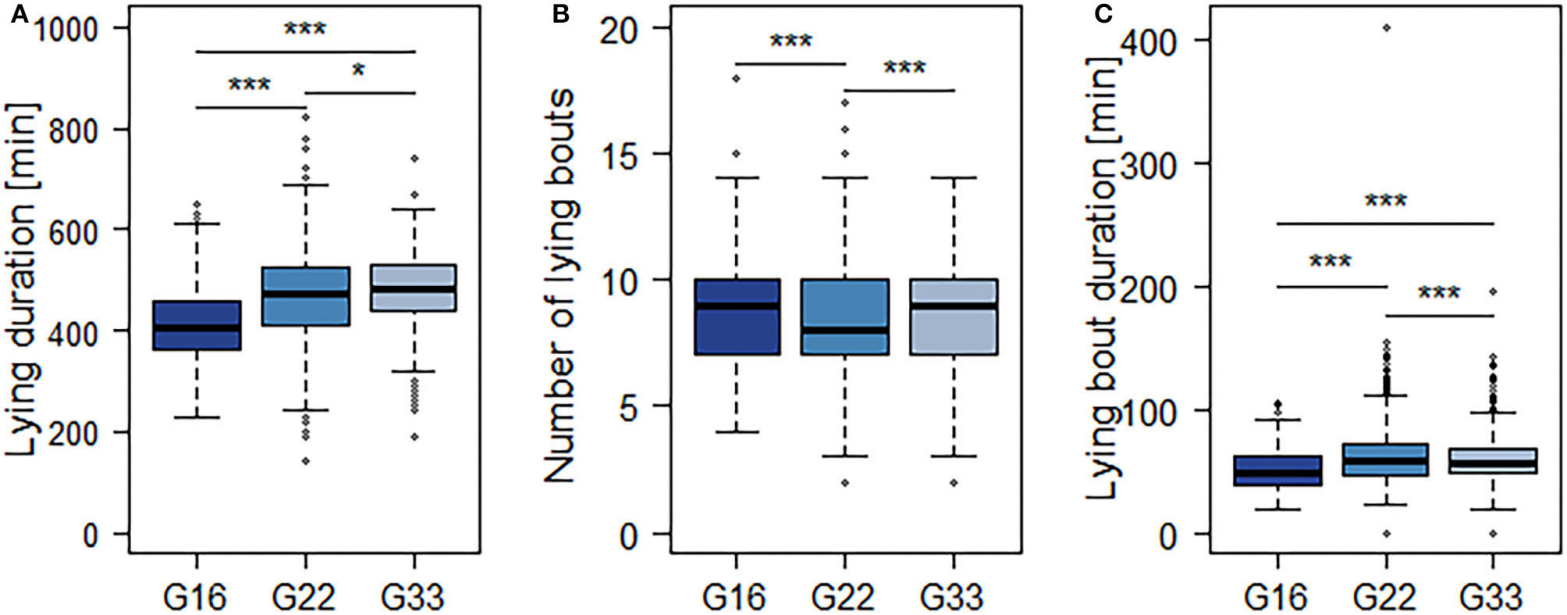
Mean duration of time spent lying **(A)**, number **(B)** and mean duration **(C)** of lying bouts per 15.5 h-period. Dark blue = groups of 16 bulls (G16), blue = groups of 22 bulls (G22), light blue = groups of 33 bulls (G33). Data were averaged for three observation periods with three 15.5 h-periods of observation each and two groups of 16 and 33 animals each as well as four groups of 22 animals. *p < 0.05; ***p < 0.001.

The averaged feeding duration per animal and 15.5 h-period varied from 127 min in G22 to 153 min in G16 (Figure 4A). Bulls in G22 on average spent less time feeding than those in G16 and G33 (both *p* < 0.0001; Table 4). Further significant differences were observed regarding the mean number and duration of feeding bouts: Bulls in G16 displayed a higher number of feeding bouts than those in larger groups (G16 vs. G22: *p* < 0.0001; G16 vs. G33: *p* = 0.0007; Figure 4B), while the mean duration of feeding bouts was higher in G33 (both *p* < 0.0001; Figure 4C). Bulls in G22 displayed slightly less feeding bouts than those in G33 (*p* = 0.0028) and slightly shorter feeding bouts than those in G16 (*p* = 0.0243). The mean duration of time bulls were lying increased with group size from 416 min in G16 to 465 min in G22 and 481 min in G33 (G16 vs. G22, G16 vs. G33: *p* < 0.0001; G22 vs. G33: *p* = 0.0338; Figure 5A). Bulls in G22 on average displayed fewer lying bouts than bulls in G16 and G33 (G16 vs. G22: *p* = 0.0003, G22 vs. G33: *p* < 0.0001; Figure 5B), and the mean lying bout duration of bulls in G16 was reduced in comparison to the larger groups (both *p* < 0.0001; Figure 5C). In G22, lying bouts were slightly longer than in G33 (*p* = 0.0009). For all of the described variables of lying and feeding behavior at individual level, standard deviation was similar for all group sizes (Figures 4, 5). Feeding duration as well as number of lying and feeding bouts and lying bout duration were not significantly correlated with carcass weight or BCS (Table 5). Significant correlations existed between mean feeding bout duration and BCS (*p* = 0.0423) and mean lying duration and carcass weight (*p* = 0.0019; Table 5). However, Spearman's rank correlation coefficients indicate weak correlations in both variables (Table 5).

**Table 5:** Spearman's correlation of the variables describing feeding and lying behavior with carcass weight and body condition score (BCS).

**Variable**	**BCS**		**Carcass weight**	
	**r_**sp**_**	***p***	**r_**sp**_**	***p***
Feeding duration (min)	0.0383	0.6031	0.0168	0.8210
Lying duration (min)	0.0631	0.3906	0.2278	0.0019
Number of feeding bouts	−0.0611	0.4064	−0.0424	0.5680
Number of lying bouts	−0.0434	0.5553	−0.0347	0.6397
Mean feeding bout length (min)	0.1486	0.0423	0.0709	0.3387
Mean lying bout length (min)	0.0694	0.3455	0.1376	0.0626

*r_sp_, Spearman's rank correlation coefficient; min, minutes. Degrees of freedom: 184*.

Over the course of the fattening period, all variables of lying and feeding behavior at individual level developed similarly in all group sizes (Supplementary Table 1): The time spent lying as well as the mean lying bout length increased with OP, while the mean number of lying bouts decreased (all *p* < 0.0001). The mean feeding duration, number and duration of feeding bouts decreased with OP (all *p* < 0.0001).

## Discussion

The aim of this study was to analyze behavior and performance of fattening bulls housed in different group sizes to gain first scientific knowledge on the suitability of large group systems for the housing of fattening cattle. Health and growth performance were satisfactory in all group sizes. Regarding health and BCS values, no differences between group sizes were observed. No severe injuries or health problems occurred, and no animals were scored with BCS values below 2.0 or higher than 4.0, indicating emaciated or obese body conditions. Furthermore, BCS values increased over the course of the fattening period, converging to a value of 3.0, which is consistent with well-balanced frame and covering. Consequently, bulls scored with a low BCS at the first OP were able to improve their body condition over the course of the fattening period, indicating they had no restricted access to feed due to their weaker body condition. Mean carcass weights were consistent with the average carcass weights of Simmental bulls in Lower Saxony (34). In G16, they were lower than in the larger groups, indicating better growth performances with increments of group size. However, it is unlikely that group size per se caused the lower carcass weight as the animals in G16 were also younger at slaughter. With 517.4 ± 10.6 days, their average age was 15 days lower than that of the bulls in G33 and even 45 days lower in comparison to those in G22. As average daily weight gain in fattening bulls reaches up to 1.4 kg, these age differences possibly influenced the carcass weights to a considerable degree (35–37). Furthermore, weight at the beginning of fattening as well as daily weight gain were not analyzed at individual level in this study and the animals housed on farm 1 (G22) were fed another diet than those on farm 2 (G16 and G33), leading to restrictions in interpreting carcass weights. However, negative effects of larger group sizes on weight gain were not observed. Further differences occurred regarding the range of carcass weights that was clearly higher in G22-animals. However, as range and SD were smaller in G16 as well as in G33, these differences are also more likely to be caused by farm management than by group size. This is confirmed by the age of the animals at slaughter, which was higher with a wider range in G22. Furthermore, the animals from farm 1 (G22) were mostly slaughtered as complete groups, while groups on farm 2 (G16 and G33) were divided into subgroups and slaughtered at various dates when animals clearly differed in body size.

### Behavioral Patterns Displayed in All Group Sizes

Regarding the animals' behavior, there were several patterns similarly displayed in all group sizes. One aspect is the distribution of the animals' activity over the course of the day. There was one main lying period during the night and early morning hours, when the averaged percentage of bulls lying reached nearly 100%, and two further lying periods with fewer animals lying simultaneously in the afternoon. An average of <20% of the animals lying was only observed during the periods of feed delivery, when maximum percentages of bulls feeding occurred. Consistent with other studies on cattle fed once or twice per day, there were clear peaks of feeding activity during and shortly after the periods of feed delivery in all group sizes (35, 38–40). These peaks were higher at morning feeding indicating that the animals were hungrier in the morning after the longer fasting period without a delivery of fresh feed during the night. Apart from these peaks, the feeding activity was widely spread over the whole daylight period from about 07:00 to 22:00 h, while being reduced during the night and early morning hours. This pattern resembles the grazing behavior cattle display under natural conditions, spreading out their feeding behavior over the whole day with several grazing periods from dawn to dusk (8, 41). Similar feeding activity was described by Cozzi et al. (42) for fattening cattle and by DeVries et al. (38) for dairy cows.

Another aspect of behavior consistent in all group sizes was the development of lying and feeding behavior over the course of the fattening period: Lying duration as well as mean lying bout length increased, while the mean number of lying bouts decreased, consistent with other studies (43–47).

Another typical trait characterizing the natural feeding behavior of cattle is synchronization (8, 48). In housed cattle, the strong desire of the animals to access the manger as a group may lead to feed competition with negative health effects for individual animals (49). Feed competition may reduce the average meal duration of cattle and dominant animals with ad libitum access to feed may eat a larger amount of dry matter than subordinate individuals (50). However, according to several authors, housed cattle display less behavioral synchronization than cattle under natural conditions (8, 51–53). In fattening cattle, one reason for this could be the common animal/feeding place ratio of 2:1 permitting simultaneous feeding of only around 50% of the animals per pen (1). In accordance with the recommendations of 75 cm feeding space for bulls weighing more than 650 kg, the manger space in the present study provided at least 7.7 (G16), 12.8 (G22), and 15.2 (G33) feeding places. However, the averaged percentage of bulls observed feeding simultaneously was mostly below 20% in all group sizes, and the bulls showed a clear preference to feed alone or in small groups. Several other authors also describe this preference in fattening cattle fed ad libitum (35, 45, 54). In addition, there are studies on non-lactating dairy cows fed ad libitum also displaying a feeding behavior widely spread out over the course of the day, with a low number of cows feeding at any time of the day (55, 56). Limitations of manger space seem unlikely to be the reason for the animals' preference to feed alone or in small groups. Firstly, this is indicated by the study by Gottardo et al. (45), in which the authors compared two different manger spaces and found the bulls' feeding behavior not to be affected by manger space. Secondly, the bulls in the present study obviously did not suffer from restricted access to feed. This is indicated by the satisfactory growth performance and BCS values. Consequently, a more likely explanation for their preference to feed alone or in small groups is that the motivation of cattle to synchronize their feeding activity is reduced by the permanent availability of feed in an ad libitum feeding regimen (45, 56). This argumentation is supported by Longenbach et al. (57) who found a strong motivation to simultaneously visit the manger in heifers with restricted feeding.

Nonetheless, interactions occurred mainly during and after feed delivery in all group sizes. Therefore, they seem to be primarily caused by feed competition. However, the averaged percentage of bulls participating in interactions was still quite low at any time of the day. Furthermore, the feeding duration of the individual bulls as well as the number of bouts displayed indicates that feeding was not restricted by an increased occurrence of aggression in any of the group sizes: With mean feeding durations of 127–153 min and 8.3–9.4 feeding bouts per 15.5 h-period, the observed feeding behavior was comparable to literature values for fattening bulls ranging from 90 to 147 min per day (35, 47, 58–60) divided into six to ten bouts (48, 59). The same applies to the lying behavior observed in the present study, with mean durations ranging from 416 to 481 min divided into eight to nine bouts. As these values refer to a 15.5 h-period excluding the night and early morning hours, when the highest percentages of animals lying were observed, they seem to be comparable to literature values fluctuating around 800 min and nine to 18 bouts per day (46, 59, 61, 62). Therefore, impaired lying behavior due to increased aggression seems unlikely.

In contrast to the feeding behavior, the observed lying behavior was highly synchronized in conformance with the natural behavior of cattle as gregarious animals (48, 63): The most frequent lying condition was an average of 81–100% of the animals per pen lying in all group sizes. Furthermore, simultaneous lying of all animals per pen occurred in all pens. In contrast, several studies on dairy cattle reported synchronization levels of more than 80% occurring only during short periods of time or not at all (55, 64, 65). Similar observations of highly synchronized lying behavior, but hardly synchronized feeding activity, exist for dairy cows (56). As an explanation for the lack of synchronization in feeding behavior, the authors of this previous study named the constant feed availability and composition of conserved feed fed ad libitum. Synchronous lying is used as an indicator for high levels of animal welfare (66, 67), and various authors agree on its importance. Galindo and Broom (68) found an increased occurrence of lameness in low ranking dairy cows that were forced to stand when synchronous lying of all animals per herd was not possible. Mogensen et al. (69) confirmed the importance of synchronous lying behavior by proving the high priority of lying synchronization in heifers: In slatted pens with a littered lying area too small to allow for simultaneous lying of all animals per pen, they observed one heifer per pen lying more frequently on the concrete slats. These heifers were characterized by lower daily weight gains. The findings of the present study emphasize the importance of simultaneous lying in fattening cattle and, therefore, the necessity of sufficient space for simultaneous lying. At least at the observed space allowances of 3.5–4.5 m^2^, simultaneous lying of all animals per pen was possible.

### Differences Between Group Sizes

In the present study, the lying behavior tended to be even more synchronized with increasing group size: Synchrony levels of 61–100% were observed more often in G33 than in smaller groups. As mentioned above, synchronous lying has a high priority in cattle and high levels of synchrony indicate high levels of animal welfare. In addition, the mean duration of time the animals spent lying clearly increased with group size, and lying bouts were longer in the larger groups of 22 and 33 animals. This observation also suggests the large groups to be beneficial as lying behavior in fattening cattle is an indicator of comfort and a possibility to promote productivity (70).

Furthermore, bulls in G22 and G33 performed on average fewer feeding bouts that had a higher average duration in the largest group size. These observations indicate increasingly undisturbed lying and feeding behavior with increments of group size and, therefore, reject the common view that increasing group size leads to increased aggression (5, 7, 8). If this was the case, increments of group size would lead to more disturbances of lying and feeding animals and, consequently, reduced synchronization. However, the observations of the present study indicate the opposite.

Consistently, the mean and maximum percentage of animals participating simultaneously in interactions was lower in large groups of 33 animals than it was in small groups of 16 animals. As there was no distinction between sociopositive interactions and aggression nor an analysis at individual level, further analysis of the effect of group size on aggression in cattle is needed to derive a reliable conclusion. However, these first results indicate that there is no linear increase in aggression with increments of group size in fattening cattle. Similarly, various authors describe a decline in aggression with increasing group size in poultry (10–13) as well as in pigs (14–16). In cattle, Kondo et al. (17) observed a linear increase in aggression with group size, but only in adult cattle older than 2 years in mixed-sex groups. Therefore, this former study is hardly comparable to the housing of fattening cattle. To our knowledge, this is the only study examining the effects of group size in large groups of cattle. Further studies refer to smaller groups of a maximum of 16 animals and they differ regarding their conclusion on the effects of group size. The study by Kondo et al. (17) also included smaller groups of two to 12 calves aged up to 13 months, where no correlation between aggression and group size could be observed. Rind and Phillips (19) compared groups of four to 16 dairy cows and observed an increased aggression in groups of 16. In contrast, Telezhenko et al. (22) found no effects on the level of aggression when housing dairy cows in groups of six or 12. In agreement, lying behavior of dairy cows housed in groups of six or 12 did not differ (18, 22), and group size had no effect on the productivity of dairy cows (18, 19).

The social tolerance hypothesis challenges the increase in aggression with group size (10): It states that larger groups with unlimited access to food and water have a more tolerant social system as it is uneconomical for individual animals to expend energy in defending resources from others (23). The more tolerant social system with few agonistic encounters, enables the animals to spend more time in exploiting the available resources (7). Therefore, access to resources in large groups should not be impaired. An observation supporting this hypothesis is the dependence of group size on the abundance of resources under natural conditions (7, 71): In habitats with high food availability, the level of competition is low and large groups can be sustained. On the contrary, if resources are limited, groups are smaller due to increased competition. Such an influence of food abundance and distribution on group size has also been observed in cattle (72). The present study also confirmed these theories, as bulls spent more time lying with increasing group size and displayed a more synchronized lying behavior as well as increasingly undisturbed lying and feeding behavior. Consistently, the mean and maximum percentages of animals participating in interactions did not increase with group size. A restriction of individual animals can also be excluded as the standard deviation of the variables describing lying and feeding behavior at individual level was similar in all group sizes. Consequently, with increments of group size, there was no increasing variation between individuals. This is confirmed by the satisfactory growth performance as well as the absence of significant strong correlations between the individual level data on feeding and lying behavior and carcass weight or BCS: Restricted access to resources or severe restrictions of individuals in lying and feeding behavior would possibly lead to impaired growth performance.

In addition to the social tolerance hypothesis, the hypothesis of shared free space also indicates large group systems to be suitable housings systems for fattening cattle. It is based on the idea that group-housed animals can share their free space, leading to larger pens providing in total more free space in which animals can move, even if the number of animals per square meter is kept constant (24, 25). In the present study, the feeding behavior was less synchronized with increasing group size, as the percentage of time without any bulls feeding decreased and was lowest in G33. This could be explained by the spatial separation of functional areas in larger groups, facilitated by the increasing amount of shared free space. Active animals can be probably found in the front part of the pen containing food and water, while lying animals are more likely to retreat to the back part of the pen. With group and pen size, the distance between the front and back part of the pen increases, possibly leading to fewer disturbances of lying animals by active ones. According to Schrader (73), this reduction in disturbances between animals displaying different activities leads to farm animal housing systems with spatially separated functional areas being behaviorally more appropriate. The reduced occurrence of disturbances with increments of group size mentioned above could confirm this hypothesis. Furthermore, allelomimetic behavior in cattle is more likely to occur between neighboring than between randomly selected animals (63). Consequently, in large groups, the greater distance between lying and feeding animals may reduce the occurrence of allelomimetic effects between them.

The animals in G22 spent significantly less time feeding than those in the smaller and larger groups. Consistently, they displayed less and shorter feeding bouts than both G16 and G33. However, this difference is more likely to be caused by differences in feed composition than by group size per se as the animals of G22 were housed on farm 1 and received another TMR than the other group sizes on farm 2. The TMRs clearly differed in particle size distribution with long particles making up 7.88% of the ration on farm 2 and only 3.87% on farm 1. As the time cattle spend feeding is known to depend on silage quality (74), the less structured feed on farm 1 (G22) may be ingested faster.

To summarize, the results of the present study indicate large group systems to be suitable for the housing of fattening cattle. This applies to G22 as well as G33. Large groups may even improve animal welfare as was indicated by an increased level of lying synchronization, higher percentages of time spent lying and a tendency to show increasingly undisturbed feeding and lying behavior. Thus, the hypotheses of large groups of fattening cattle having a more tolerant social system and facilitation of spatial separation of functional areas in large groups were confirmed. Consequently, the observations of the present study do not provide scientific evidence to support the existing housing recommendations for fattening cattle with maximum group sizes of 12–20 animals (2–4). On the contrary, the indication of positive influences of increments in group size on animal welfare confirms SCAHAW (5) in claiming that more information on maximum as well as optimum group size in cattle is required. Further studies analyzing the effect of group size on aggression in fattening cattle are needed to offer reliable conclusions on suitable group sizes. Additionally, the economic dimensions of housing fattening bulls in large groups should be evaluated in detail. The higher carcass weights in larger groups observed in this study could also be a result of management factors. Therefore, further studies should include control groups housed in exactly the same housing systems with consistent management to avoid effects of group size being hidden by feed or other management factors. Furthermore, daily weight gain should be monitored at individual level for more detailed information on possible effects on growth performance. Another aspect, further studies should consider is the human-animal relationship, e.g., by performing approach/avoidance tests. According to Waiblinger et al. (75), an increased understanding of the human-animal-relationship is an essential component of strategies intending to improve animal welfare. This also applies to large group housing systems, with regard to animal welfare as well as the farmers' safety. The handling of the bulls may differ depending on group size and may be even aggravated in large groups. Consistently, EFSA (6) states that the effect of group size on the quality of the interactions between humans and cattle should be further studied. In addition, further studies should analyze the ruminating behavior of the bulls as it may be associated with lying and feeding and changes in rumination time are reliable and early indicators of health problems in cattle (50, 76). Another limitation of the study is that all observations were performed by a single observer. Consequently, observer effects cannot be fully excluded.

The importance of further research on group size is stressed by the fact that housed farm animals may suffer from negative effects provoked by housing in unsuitable group sizes, as they lack the natural possibility of leaving a group depending on environmental conditions (7). Therefore, there is an urgent need to review the suitability of common group sizes and consider the results in order to revise existing housing recommendations and introduce obligatory regulations.

## Conclusion

Large group systems with 22–33 animals are suitable for the housing of fattening cattle and may even improve animal welfare. With increments of group size, lying behavior was more synchronized and the animals spent more time lying, displaying a tendency to increasingly undisturbed feeding and lying behavior. Furthermore, the mean and maximum percentages of animals participating simultaneously in interactions as well as interindividual variations in lying and feeding behavior did not increase with group size. These observations provide no scientific evidence for the view that increasing group size leads to increased aggression and they do not scientifically support existing housing recommendations for fattening bulls with group sizes of at maximum 12–20 animals. Consequently, these recommendations should be revised and the effect of group size on behavior and growth performance of fattening cattle should be analyzed further.

## Data Availability Statement

The datasets generated for this study are available on request to the corresponding author.

## Ethics Statement

The animal study was reviewed and approved by Animal Welfare Officer of the University of Veterinary Medicine Hannover, Foundation. For this study, no ethical approval had to be obtained because it neither involved a prospective evaluation nor laboratory animals and only non-invasive procedures were applied.

## Author Contributions

LS and BS contributed to conception and design of the study. LS organized the database and wrote the first draft of the manuscript. LS, NV, NK, and BS performed the statistical analysis. NV, NK, and BS reviewed and edited the manuscript. All authors contributed to manuscript revision, read and approved the submitted version.

## Conflict of Interest

The authors declare that the research was conducted in the absence of any commercial or financial relationships that could be construed as a potential conflict of interest.
