# Supplementary material for: Large Group Housing Systems in Fattening Bulls—Comparison of Behavior and Performance
Source: Front Vet Sci. 2020 Dec 9;7:543335. doi: 10.3389/fvets.2020.543335 (PMC7756093; doi:10.3389/fvets.2020.543335)
Supplement: Supplementary file 1 [file Data_Sheet_1.docx]

Supplementary Material

# Supplementary Table

**Supplementary Table 1.** Variables describing lying and feeding behavior in fattening bulls with differentiation towards observation period (OP) and p- and t-value statistic of Bonferroni adjustment for multiple comparisons. G16 = groups of 16 animals, G22 = groups of 22 animals, G33 = groups of 33 animals, min = minutes, DF = degrees of freedom, dur. = duration.

| **Variable** | **Group size** | **OP1** | **OP2** | **OP3** | **DF** | **t** | **p** |
| --- | --- | --- | --- | --- | --- | --- | --- |
| Lying duration [min] | G16 | 369.4 ± 58.9 | 414.6 ± 56.0 | 464.0 ± 99.2 | 1,567 | 85.1 | < 0.0001 |
|  | G22 | 427.5 ± 89.5 | 479.1 ± 96.8 | 487.7 ± 93.8 | 1,567 | 158.1 | < 0.0001 |
|  | G33 | 455.6 ± 62.8 | 488.2 ± 88.2 | 486.7 ± 54.2 | 1,567 | 126.1 | < 0.0001 |
| Number of lying bouts | G16 | 10.2 ± 2.2 | 8.0 ± 1.91 | 8.1 ± 2.0 | 1,567 | 65.2 | < 0.0001 |
|  | G22 | 8.8 ± 2.4 | 8.2 ± 2.65 | 7.5 ± 2.2 | 1,567 | 101.5 | < 0.0001 |
|  | G33 | 8.9 ± 2.2 | 8.9 ± 1.84 | 8.0 ± 2.0 | 1,567 | 81.4 | < 0.0001 |
| Lying bout dur. [min] | G16 | 3.7 ± 0.9 | 5.5 ± 1.48 | 6.0 ± 1.6 | 1,567 | 38.5 | < 0.0001 |
|  | G22 | 5.2 ± 1.7 | 6.6 ± 3.53 | 7.1 ± 2.9 | 1,567 | 78.1 | < 0.0001 |
|  | G33 | 5.5 ± 2.1 | 5.8 ± 1.76 | 6.5 ± 2.0 | 1,567 | 58.2 | < 0.0001 |
| Feeding duration [min] | G16 | 228.4 ± 65.4 | 122.2 ± 29.9 | 107.60 ± 25.2 | 1,567 | 49.7 | < 0.0001 |
|  | G22 | 152.9 ± 45.1 | 121.0 ± 42.5 | 108.2 ± 37.3 | 1,567 | 69.2 | < 0.0001 |
|  | G33 | 199.7 ± 55.9 | 145.7 ± 44.6 | 123.0 ± 32.0 | 1,567 | 61.3 | < 0.0001 |
| Number feeding bouts | G16 | 11.9 ± 2.5 | 8.3 ± 2.0 | 8.0 ± 1.8 | 1,567 | 63.9 | < 0.0001 |
|  | G22 | 9.4 ± 2.3 | 8.3 ± 2.6 | 7.4 ± 2.1 | 1,567 | 95.1 | < 0.0001 |
|  | G33 | 10.3 ± 2.7 | 8.4 ± 2.2 | 7.7 ± 1.9 | 1,567 | 74.0 | < 0.0001 |
| Feeding bout dur. [min] | G16 | 2.0 ± 0.5 | 1.5 ± 0.3 | 1.4 ± 0.3 | 1,567 | 63.2 | < 0.0001 |
|  | G22 | 1.7 ± 0.4 | 1.5 ± 0.3 | 1.5 ± 0.4 | 1,567 | 100.9 | < 0.0001 |
|  | G33 | 2.0 ± 0.6 | 1.8 ± 0.5 | 1.6 ± 0.4 | 1,567 | 89.3 | < 0.0001 |

# Supplementary Figure


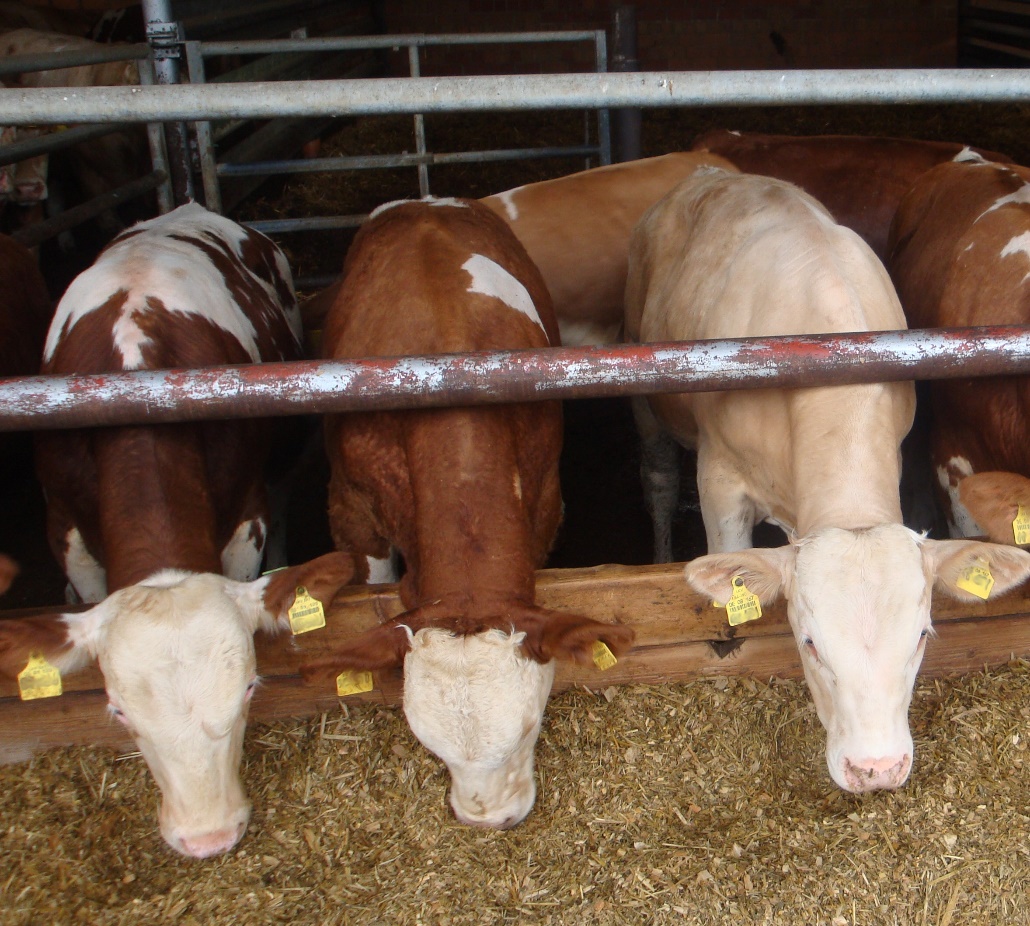


**Supplementary Figure 1.** Feeding area with horizontal metal tubes.
